# Honey Targets Ribosome Biogenesis Components to Suppress the Growth of Human Pancreatic Cancer Cells

**DOI:** 10.3390/cancers16193431

**Published:** 2024-10-09

**Authors:** Aun Ali Bangash, Sahir Sultan Alvi, Muhammad Ali Bangash, Haider Ahsan, Shiza Khan, Rida Shareef, Georgina Villanueva, Divyam Bansal, Mudassier Ahmad, Dae Joon Kim, Subhash C. Chauhan, Bilal Bin Hafeez

**Affiliations:** 1 South Texas Center of Excellence for Cancer Research, School of Medicine, University of Texas Rio Grande Valley, McAllen, TX 78504, USA; aun.bangash01@utrgv.edu (A.A.B.); muhammad.bangash01@utrgv.edu (M.A.B.); haider.ahsan01@utrgv.edu (H.A.); shiza.khan01@utrgv.edu (S.K.); rida.shareef01@utrgv.edu (R.S.); georgina.villanueva01@utrgv.edu (G.V.); mudassier.ahmad@utrgv.edu (M.A.); dae.kim@utrgv.edu (D.J.K.); subhash.chauhan@utrgv.edu (S.C.C.); 2 Department of Medicine and Oncology ISU, Division of Immunology and Microbiology, School of Medicine, University of Texas Rio Grande Valley, McAllen, TX 78504, USA; 3 Department of Kinesiology, Rice University, Houston, TX 77251, USA; db65@rice.edu

**Keywords:** pancreatic cancer (PanCa), ribosome biogenesis, honey, UBTF, RPA194, RPA135, nucleolar stress, apoptosis, proliferation

## Abstract

**Simple Summary:**

Pancreatic cancer (PanCa) is one of the deadliest forms of cancer with limited therapeutic options. The available conventional therapies are highly toxic and often show resistance. Accumulating evidence suggests that dysregulated ribosome biogenesis has been linked to the survival and aggressive phenotypes of many tumor types, including PanCa. Thus, targeting ribosome biogenesis could be a novel approach for suppressing the growth of PanCa. The current study aimed to investigate the anti-cancer efficacy and underlying molecular mechanisms of honey against PanCa. Our results demonstrated that honey induces apoptosis and inhibits the growth and invasive potential of PanCa cells by targeting ribosome biogenesis components and c-Myc expression. This study suggests that honey can be used as an adjuvant along with conventional chemo/radiation therapy or immunotherapy for the management of PanCa.

**Abstract:**

Pancreatic cancer (PanCa) is one of the deadliest cancers, with limited therapeutic response. Various molecular oncogenic events, including dysregulation of ribosome biogenesis, are linked to the induction, progression, and metastasis of PanCa. Thus, the discovery of new therapies suppressing these oncogenic events and ribosome biogenesis could be a novel therapeutic approach for the prevention and treatment of PanCa. The current study was designed to investigate the anti-cancer effect of honey against PanCa. Our results indicated that honey markedly inhibited the growth and invasive characteristics of pancreatic cancer cells by suppressing the mRNA expression and protein levels of key components of ribosome biogenesis, including RNA Pol-I subunits (RPA194 and RPA135) along with its transcriptional regulators, i.e., UBTF and c-Myc. Honey also induced nucleolar stress in PanCa cells by reducing the expression of various nucleolar proteins (NCL, FBL, and NPM). Honey-mediated regulation on ribosome biogenesis components and nucleolar organization-associated proteins significantly arrested the cell cycle in the G2M phase and induced apoptosis in PanCa cells. These results, for the first time, demonstrated that honey, being a natural remedy, has the potential to induce apoptosis and inhibit the growth and metastatic phenotypes of PanCa by targeting ribosome biogenesis.

## 1. Introduction

Pancreatic cancer (PanCa) is considered one of the deadliest cancers, having substantially lower early detection rate, rapid advancement, and highly prone to develop chemoresistance [1,2]. The five-year survival for PanCa is as low as only 10–12%, which makes it the third leading cause of cancer-associated casualties [3], and it has been speculated that it will become the second leading cause of such deaths (~46,000–50,000 annually) in the United States by the year 2040 [4]. Distinct risk factors have been associated with the initiation, progression, advancement, and drug resistance in PanCa, including age, sex, pathological staging, tumor microenvironment, epigenetic alterations, immune landscape, various signaling pathways, and single or multiple genetic aberrations in KRAS, T53, SMAD4, and cyclin-dependent kinase inhibitor 2A (CDKN2A) [5,6]. Recent research and clinical trials across the globe have established gemcitabine and a combination of oxaliplatin, irinotecan, fluorouracil, and leucovorin (FOLFIRINOX) as first-line standard therapeutic regimens, where the latter has an advantage over gemcitabine in terms of median overall survival (11.1 months vs. 6.8 months, respectively) [7]. Despite better therapeutic efficacy, FOLFIRINOX therapy has been linked to the emergence of severe toxicity in patients with PanCa, leaving no choice other than using gemcitabine with nab-paclitaxel as a key therapeutic option in clinical settings [1,2]. However, the development of resistance against gemcitabine has challenged the use of this standard-of-care therapy and made it alarming to discover alternative molecular targets and develop respective specific targeting modalities for PanCa.

Unlike normal cells, metabolically active cancer cells require a relatively higher pace of protein synthesis, which is met by the aberrant regulation of ribosome biogenesis, a key process that regulates the synthesis of ribosomes [8]. The multistep ribosome biogenesis occurs in the nucleolus, where RNA Pol-I transcribes rDNA into pre-ribosomal RNA [8]. The processivity of RNA Pol-I is coordinately regulated by two of its catalytic units, including RPA194 (encoded by the gene POLR1A) and RPA135 (encoded by the gene POLR1B) [9,10]. On the other hand, upstream binding transcription factor (UBTF), a member of the HMG-box DNA-binding protein family and an integral part of ribosome biogenesis, has been linked to the advancement and chemoresistance in cancer [11]. UBTF facilitates the assembly of the transcription pre-initiation complex (PIC) of RNA Pol-I on the promoter and the establishment of potentially active rDNA chromatin conformation. UBTF binds to rDNA enhancer/promotor and cooperates with the multi-component SL1 transcription factor complex, composed of the TATA-binding protein (TBP) and TAF factors A-D, to form the PIC assembly and subsequent rDNA transcription [12]. Interestingly, the pleiotropic regulatory role of UBTF can be understood as it impacts both upstream and downstream ribosome biogenesis events to orchestrate rDNA transcription [12]. Owing to its detrimental role in actively proliferating cancer cells, various studies have speculated that targeting UBTF might become a promising therapeutic strategy in the management of cancer [11,13].

Apart from the key catalytic units of RNA Pol-I (i.e., RPA194 and RPA135) and their transcriptional activator (i.e., UBTF), various ribosomal proteins (RPs) that are known to form structural counterparts of ribosomes also play a critical role in ribosome biogenesis [14]. Genetic mutations and aberrant expression of the RPs (such as RPS23, RPS27, RPL29, and RPL35) have been linked to various cancers [14,15]. Recently, various specific inhibitors have been tested for their ability to target RNA Pol-I activity, including aclarubicin and Curaxin CBL0137. These inhibitors affect chromatin biology and interfere with the chromatin stability of all three RNA Pol (namely, RNA Pol-I, II, and III) [16]. Further, researchers have revealed an unanticipated ability of selective Pol-I inhibitors to stabilize DNA G-quadruplex and induce DNA damage in cancerous cells, but its activity depends upon the deficiency of BRCA1/2, which ultimately leads to the compromised homologous recombination and non-homologous end joining DNA repair system [17]. To date, no natural pharmacological inhibitor has been developed for the effective and selective targeting of ribosome biogenesis components, including UBTF and RPA194, along with RPs, to treat cancer due to their nucleolar localization and other unfavorable surface attributes. These findings have clearly highlighted the unmet need for the discovery of an alternative natural therapeutic regimen targeting ribosome biogenesis for the treatment of various malignancies, including PanCa. Different natural remedies and secondary metabolites have been used traditionally for the management of various health complications, including cancer [18], diabetes [19], cardiovascular diseases [20,21,22], neurological disorders and protein aggregation [23,24], infectious diseases [25], and oxidative stress [26].

**Figure 1:**
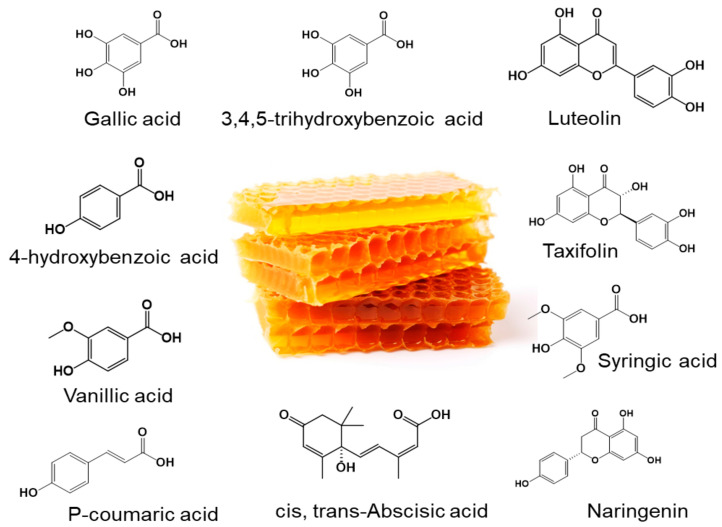
Schematic representation of natural metabolites of honey. The image of the honeycomb, which is free to download and use, was downloaded from https://www.vecteezy.com/ (accessed on 1 October 2024).

In the same context, honey, a natural remedy made by honeybees through a complex mechanism, has been used by different traditional medicine systems across the globe [27]. It is well-known for its beneficial pharmacological effects against various diseases, including but not limited to cancer [28], cardiovascular diseases [29], oxidative stress [30,31], wound healing [32], anti-inflammatory diseases [33,34,35], diabetes [36,37], neurodegenerative diseases [38], and gastrointestinal diseases [39]. A recent study has shown its potential therapeutic efficacy against triple-negative breast cancer via the activation of AMPK and inhibition of AKT/mTOR downstream signaling in MCF-7 cells [40]. It also reduced the level of phosphorylated STAT3 in vitro, along with a marked regression (~84%) in MCF-7 cells-derived tumors in immunocompromised mice [40]. Another study demonstrated that honey enhances the sensitivity of HepG2 cells for doxorubicin and induces apoptosis by targeting Wnt/β-catenin and ERK1/2 signaling pathways, while no toxicity was reported in normal hepatic cells [41]. Moreover, different Polish honeys have been shown to inhibit the proliferation and metastasis in human glioblastoma multiforme U87MG cells by inhibiting matrix metalloproteinases (MMP-2 and MMP-9) [42]. The pharmacological effects of honey have been attributed to its bioactive secondary metabolites, i.e., gallic acid, ferulic acid, 4-hydroxyquinoline, caffeic acid, chlorogenic acid, catechin, kynurenic acid, chrysin, myricetin, syringic acid, abscisic acid, and kaempferol (Figure 1) [28]. However, the anti-cancer potential of honey against PanCa via targeting ribosome biogenesis components has remained elusive. Therefore, the current study was designed to delineate the anti-cancer potential of honey against PanCa. We have demonstrated the effect of honey on various components of ribosome biogenesis (UBTF, RPA194, and RPA135), RPs, c-Myc, nucleolar proteins (NCL, FBL, and NPM), apoptosis, and cell cycle analysis in PanCa cells.

## 2. Materials and Methods

### 2.1. Materials

The DMEM-high glucose Medium (Cat. No. #11965092), RPMI 1640 Medium (Cat. No. #11875093), DMEM/F-12 Medium (Cat. No. #11320033), McCoy’s 5A (Modified) Medium (Cat. No. #16600082), Fetal Bovine Serum (FBS) (Cat. No. #10437-028), Trypsin-EDTA (0.25%) with phenol red (Cat. No. #25200056), Phosphate buffer saline (PBS)-pH 7.4 (Cat. No. #10010023), and Antibiotic-Antimycotic (100X) (Cat. No. #15240062) were procured from Gibco^TM^ by Life Technologies, Carlsbad, CA, USA. The Invitrogen™ dead cell apoptosis kit with annexin V for flow cytometry (Cat. No. #V13241), FxCycle™ PI/RNase staining solution (Cat. No. # F10797), and TRIzol™ Reagent (Cat. No. #15596018) were obtained from Thermo Fisher Scientific, Waltham, MA, USA. MTT (Cat. No. # 475989) was obtained from Sigma-Aldrich, Burlington, MA, USA. The Kelley honey was procured from Sam’s Club, Brownsville, TX, USA.

### 2.2. Cell Culture

The MIA PaCa-2 (Cat. No. #CRM-CRL-1420), AsPC-1 (Cat. No. #CRL-1682), Capan-2 (Cat. No. #HTB-80), and HPAF-II (Cat. No. #CRL-1997) cells were obtained from the American Type Culture Collection (ATCC), Manassas, VA, USA. These cell lines harbor different mutations in KRAS (G12c, 12 Asp, and 12 Val), TP53 (R248W, 135 Δ1 bp, 151 Ser, intron 4 Δ200 bp splice site), and CDKN2A/p16 (Δ 2 bp, Δ 20–25, Δ26–27, Δ 29–34, and homozygous deletions encompassing exons 1–3, and several insertions) [43]. These are the most prevalent mutations that lead to the poor prognosis of PanCa [6]. The PanCa cells were maintained in the above-specified DMEM, RPMI 1640, McCoy’s 5A, or DMEM/F-12 medium, respectively, under a humidified atmosphere at 37 °C and 5% CO_2_. The medium for each cell line was supplemented with 10% FBS and antibiotic-antimycotic solution (1X) [44].

### 2.3. MTT Assay

The effect of honey on the survival of human pancreatic cancer cells (MIA PaCa-2, AsPC-1, Capan-2, and HPAF-II) was analyzed using an MTT assay [45]. The cells were seeded in 96-well plates at a seeding density of 5 × 10^3^ cells/well in 100 µL media and incubated at 5% CO_2_ and at 37 °C to achieve 70 to 80% confluency. The different concentrations of honey (0–10% *w*/*v*) were prepared through serial dilution and added to the cells, while 100 µL fresh medium was added to the untreated control wells, followed by incubation for 24 h under standard conditions. After the incubation, the drug was removed, and 0.2 mL of MTT (20% *w*/*v*, prepared in the respective medium; MTT stock, 5 mg/mL in PBS- pH 7.4) was added to each well and incubated for 2 to 4 h. The MTT-containing media was gently removed from the wells using the vacuum pump at low-pressure settings, and the formazan crystals were dissolved in 150 µL DMSO by keeping the plate on a shaker for 10 min. Absorbance was recorded on Varioskan^TM^ Lux Multimode Microplate Reader (Thermo Scientific^TM^, Waltham, MA, USA) at 570 nm. The absorbance of control wells was taken as 100%, and results were represented as percent viability with respect to control wells (±SEM). IC_50_ values were calculated by using logarithmic graphs [46].

### 2.4. Colony Formation Assay

The colony formation assay (CFA) was conducted to assess the efficacy of honey to inhibit the clonogenic ability of two PanCa cell lines, namely MIA PaCa-2 and AsPC-1. These cell lines were chosen based on the IC_50_ values obtained from the MTT assay in our study. The CFA was performed using a standard protocol described previously [47]. Briefly, exponentially growing cells were harvested using trypsinization and counted, and 200 cells from each cell line were seeded in triplicate in six-well plates. The cells were then allowed to adhere to the surface of the wells. After appropriate incubation (2–3 days), the cells were treated with different concentrations of honey (0, 0.5, and 0.75%; *w*/*v*) and left to form colonies (~7 to 10 days), whereas fresh medium was added to the cells in control wells. The plates were monitored under a brightfield microscope daily until colonies of the appropriate size were detected. The medium, with or without honey, was removed from the wells once the colonies were detected, and the wells were washed with PBS to remove any residual medium. The cells were then incubated with 2 mL of a mixture of 6.0% glutaraldehyde and 0.5% crystal violet and left undisturbed for 30 min. This glutaraldehyde-crystal violet solution was removed after incubation, and wells were carefully rinsed by sinking the plate into a tray filled with tap water. The colony-containing plates were then left to dry at room temperature, and plates were imaged by using Bio-Rad’s ChemiDoc™ Imaging System (Bio-Rad, Hercules, CA, USA).

### 2.5. Wound-Healing Assay

The wound-healing assay was performed to determine the migration of pancreatic cancer cells in the presence or absence of honey after an artificial wound or scratch was generated in a monolayer of cells [48]. Briefly, MIA PaCa-2 and AsPC-1 cells were seeded at 0.2 × 10^6^ cells/well in six-well plates and incubated at 37 °C and 5% CO_2_-conditioned atmosphere to achieve appropriate confluency. Once the desired confluency was achieved, a scratch was made in the center of each well using a 200 µL tip, and scratched cells were washed with PBS. Subsequently, different doses of honey (0 and 3%) in respective media were added to the wells (in triplicate), while fresh media without honey was added to the control wells. The migration ability of cells, in the absence or presence of honey, was determined at 0, 24, and 48 h time points by observing the wells under EVOS M7000 Cell Imaging System by Thermo Fisher Scientific, USA, and images were captured. The impact of honey on wound closure was determined through GraphPad Prism (version 10.0.0), and % wound closure was calculated using the following formula:

$$\%\;Wound\;closure=\left(\frac{A_{t0}-A_{\Delta t}}{A_{t0}}\right)\times 100$$

where *A_t_*_0_ is the initial wound area, and *A_Δt_* is the wound area after 24 or 48 h of the initial scratch and subsequent honey exposure.

### 2.6. Gene Expression Analysis

#### 2.6.1. RNA Isolation

For the isolation of mRNA, 1.0 × 10^6^ cells were seeded in 100 mm dishes and were treated with 5 and 7.5% honey when attained 70 to 80% confluency. After 24 h of incubation, cells were washed with cold PBS, and RNA lysates were harvested in 1 mL of TRIzol^TM^ RNA isolation reagent. The lysates were then vortexed and left at room temperature (RT) for 5 min. Following the incubation, RNA was isolated using the phenol-chloroform extraction method. Phase separation was achieved by adding 0.2 mL chloroform to each tube, and samples were incubated at RT for 2 to 3 min, followed by centrifugation at 12,000× *g* for 15 min at 4 °C. Aqueous phase was transferred to fresh tubes, and 0.5 mL isopropyl alcohol was added to each tube and incubated at RT for 10 min for RNA precipitation. The samples were centrifuged, and the retained pellet in each tube was washed with 1 mL of 75% ethanol (centrifugation at 7500× *g* for 5 min). Samples were air-dried at RT for 8 to 10 min and dissolved in equal volumes of nuclease-free water (20 µL). The concentration and purity of RNA were measured using the A260/A280 ratio with the NanoDrop Microvolume spectrophotometer, Thermo Scientific^TM^, USA. The samples with an A260/A280 ratio between 1.8 and 2.0 were used for cDNA synthesis.

#### 2.6.2. cDNA Synthesis

The high-capacity cDNA reverse transcription kit from Applied Biosystems^TM^, Waltham, MA, USA (Cat. No. #4368814) was used for the synthesis of cDNA on BioRad’s T100 Thermal Cycler. A 40 µL reaction was set up for reverse transcription of 2 µg RNA from control and treated groups. The conditions for reverse transcription were set as per kit guidelines (Step 1: 25 °C for 10 min, Step 2: 37 °C for 120 min, and Step 3: 85 °C for 5 min and infinite hold at 4 °C). The resulting cDNA was stored at −80 °C until further analysis.

#### 2.6.3. Quantitative Real-Time PCR (qRT-PCR) Analysis

The cDNA samples were used for mRNA expression through qRT-PCR analysis. Briefly, 5 µL of Bio-Rad’s SYBR green dye iQ^TM^ SYBR^®^ Green Supermix (2X), Cat. No. #170-8882), 1 µL of primer mix (stock concentration of primer mix = 10 µM), 2 µL of nuclease-free water, and 2 µL of cDNA were used to set up a 10 µL qRT-PCR reaction. As mentioned, iQ^TM^ SYBR^®^ Green Supermix contains dNTPs, iTaq™ DNA polymerase, MgCl_2_, SYBR^®^ Green I, enhancers, stabilizers, and fluorescein. The gene-specific primers used in this study are listed in Table 1. The Bio-Rad CFX96 Real-Time PCR Detection System was used for the qRT-PCR analysis using the following thermal conditions:

Polymerase activation and DNA denaturation were performed at 95 °C for 3 min. Amplification was performed for 40 cycles, involving denaturation at 95 °C for 10 s annealing/extension at 55 °C for 60 s. The data were analyzed using the 2^−ΔΔCt^ method, and change in mRNA expression was represented as a fold of control while β-actin was used as the reference housekeeping gene.

### 2.7. Western Blotting

The effect of honey on the protein expression pattern was investigated using western blotting. Briefly, 1.0 × 10^6^ million cells (MIA PaCa-2 and AsPC-1) were seeded in 100 mm dishes and allowed to reach 70 to 80% confluency, followed by the addition of honey (0, 5, and 7.5%; *w*/*v*). After 24 h of incubation, the dishes were washed with ice-cold PBS (pH 7.4), and lysates were harvested using 100 µL RIPA buffer supplemented with protease inhibitors. The lysates were sonicated at high settings (70% power rate, 10 s on/30 s off, three to five cycles), and debris was removed by centrifugation (14,000× *g* for 15 min). Protein estimation was performed using Pierce^TM^ BCA Protein Assay (Thermo Scientific, USA, Cat. No. # 23227), and an equal amount of protein (20 µg) from each group was denatured at 95 °C for 5 min in Laemmli buffer (Bio-Rad, Cat. No. #1610747) freshly supplemented with β-mercaptoethanol. The samples were resolved using Bio Rad’s pre-casted gels (4–15%) at 80 V. The resolved proteins were transferred to the PVDF membranes at 25 V for 7 min using a Trans-Blot turbo transfer system (Bio-Rad, USA) and blocked for 2 h in a 5% blocking buffer (non-fat dry milk).

The membranes were probed overnight with primary antibodies at 4 °C with gentle rocking (~20 rpm). The primary antibodies used in this study were UBTF (Santacruz, Dallas, TX, USA; #Sc-13125; 1:000), RPA194/POLR1A (Santacruz; #Sc-48385; 1:000), RPA135/POLR1B (Thermo Fisher; #PA5-39139; 1:000), c-Myc (Santacruz; #Sc-40; 1:000), RPL29 (Abcam; #ab88514; 1:000), NCL (CST; #14574S; 1:000), FBL (CST; #2639S; 1:000), NPM (Thermo Fisher; #PA5-12447; 1:000), p53 (DO-1) (Santacruz; #Sc-126; 2:000), Bcl-2 (Santacruz; #Sc-7382; 1:000), caspase-3 (CST; #9661S; 1:000), PARP (CST; #9542S; 1:000), and β-actin (Santacruz; #Sc-47778; 2:000). After this, the unbound primary antibodies were washed thrice with TBST (20 min each) and further probed either with anti-mouse IgG (H + L) HRP conjugate (Promega, Madison, WI, USA; #W402B; 1:2000) or anti-rabbit IgG (H + L) HRP conjugate (Promega; #W401B; 1:2000) for 1 h at RT. After washing out the unbound HRP conjugates, the membranes were developed using the MilliporeSigma™ Immobilon™ Western Chemiluminescent HRP Substrate (ECL) (Millipore, Billerica, MA, USA, Cat. No. #WBKLS0500) on ChemiDoc Imager (BioRad, Cat. No. #170830). The membranes were stripped using Thermo Scientific™ Restore™ Western Blot Stripping Buffer (Thermo Scientific, Cat. No. #21063) for 50 min before probing with loading controls.

### 2.8. Apoptosis Assay

The induction of apoptosis by honey was studied with flow cytometry using the Annexin V Alexa Fluor 488 & Propidium Iodide kit as per the manufacturer’s instructions. Briefly, the MIA PaCa-2 and AsPC-1 cells were seeded in 100 mm dishes at a density of 1.0 × 10^6^ cells in respective media and treated with honey for 24 h when attained 70 to 80% confluency. After completion of incubation, the cells were washed with PBS, trypsinized and centrifuged at 4000 rpm for 7 min to obtain the pellet. The pelleted cells were washed twice with ice-cold PBS and suspended in 1X annexin-binding buffer (200 to 300 µL). After this, for every 100 µL of cell suspension, 5 µL of Alexa Fluor 488 Annexin V and 1 µL of 100 µg/mL PI were added. The samples were incubated in the dark at RT for 15 min. A total of 400 µL of 1X annexin-binding buffer was added to each sample and placed on ice. The stained cells were analyzed at SONY’s ID7000™ Spectral Cell Analyzer using a 488 nm laser line and Excitation/Emission at 499, 535/521, 617 nm. The data generated were analyzed using the ID7000 system software by SONY.

### 2.9. Cell Cycle Analysis

The effect of honey on various phases of the cell cycle was assessed in MIA PaCa-2 and AsPC-1 cells. Briefly, the cells were seeded in 100 mm dishes at a density of 1.0 × 10^6^ cells in respective media and treated with honey for 24 h when 70 to 80% confluency was attained. After the desired incubation, the cells were washed with PBS, trypsinized for 3 to 5 min, and centrifuged at 4000 rpm for 7 min. The pellet was washed twice with ice-cold PBS and fixed with 70% ice-cold ethanol for 30 min or overnight at 4 °C. The cells were pelleted, washed again with PBS, resuspended in 1.0 mL FxCycle^TM^ PI/RNase solution, and incubated for 30 min at RT in the dark. The samples were analyzed with SONY’s ID7000™ Spectral Cell Analyzer using a 561 nm excitation laser and a 585/16 nm emission filter. The data generated were analyzed using the ID7000 system software by SONY.

### 2.10. Confocal Microscopy

The effect of honey on the expression of various proteins involved in ribosome biogenesis and apoptosis was investigated using confocal microscopy. The MIA PaCa-2 and AsPC-1 cells (0.04 × 10^6^/well) were seeded in 24-well plates on sterile Corning^®^ BioCoat^®^ Poly-L-Lysine glass coverslips. Different doses of honey were added to the cells and incubated for 24 h. The media were removed, and cells were washed twice with ice-chilled PBS (pH 7.4); the cells were then fixed with 4% formaldehyde for 15 min and washed with PBS to remove any residual formaldehyde. The 0.25% Triton X-100 was then added into each well for 10 min and washed again with PBS, followed by blocking with 1.0% BSA (prepared in PBS; pH 7.4) for 60 min. After blocking, primary antibodies, i.e., RPA194 (1:100), UBTF (1:200), c-Myc (1:100), NCL (1:200), and FBL (1:200) were added and left overnight at 4 °C. The cells were washed thrice with PBS for 5 min each and incubated with fluorochrome-labeled anti-mouse [Goat Anti-Mouse IgG (H + L) Secondary Antibody, FITC from Invitrogen, San Diego, CA, USA; #62-6511; 1:200] or anti-rabbit [Alexa Fluor^®^ 647 AffiniPure™ F(ab’)_2_ Fragment Donkey Anti-Rabbit IgG (H + L) from Jackson ImmunoResearch Laboratories, West Grove, PA, USA; #711-606-152; 1:100] secondary antibodies in the dark for 1 h at RT. The wells were rinsed thrice with PBS, and coverslips were mounted on glass slides using VECTASHIELD Vibrance^®^ Antifade Mounting Medium with DAPI (Cat. No. #H-1800-2); the slides were left at RT for 15 min for hardening and then stored at 4 °C in dark boxes. The images were acquired on Leica STELLARIS STED & STELLARIS 8 STED Microscope, Leica Microsystems, Wetzlar, Germany.

### 2.11. Statistical Analysis

All the experiments were conducted in triplicate, and data were represented as mean ± SEM. The statistical significance was evaluated using a two-tailed unpaired *t*-test and ANOVA, followed by Dunnett’s multiple comparison tests (whichever was applicable) through GraphPad Prism version 10.0.0 for Windows (GraphPad Software Inc., San Diego, CA, USA) [49]. A *p* value of less than 0.05 (*p* < 0.05) was considered significant; however, the level of significance for each figure was represented in respective figure legends.

## 3. Results

### 3.1. Honey Inhibits the Growth of Pancreatic Cancer Cells

**Figure 2:**
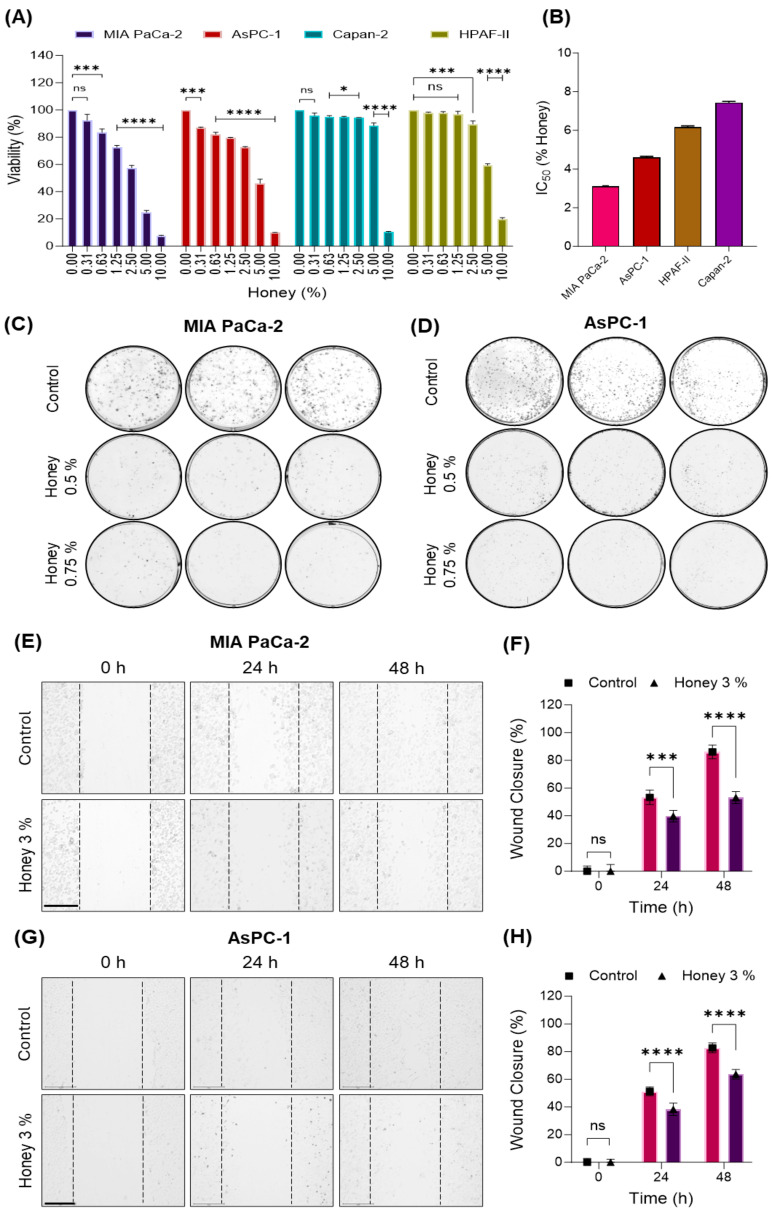
**Honey inhibits the growth and metastatic phenotype of PanCa cells**. (**A**). Effect of honey treatment on the viability of MIA PaCa-2, AsPC-1, Capan-2, and HPAF-II cells (24 h). The data are represented as the % viability ± SEM of three replicates of each dose of honey. The level of significance was calculated with respect to untreated control for each cell line. Level of significance: ^ns^ *p* ≥ 0.05; * *p* < 0.01; *** *p* < 0.0001; **** *p* < 0.0001. (**B**). IC_50_ of honey in various PanCa cell lineages; represented as the % honey ± SEM of three replicates for each cell line. (**C**,**D**). Honey reduces the clonogenic ability of MIA PaCa-2 and AsPC-1 cells. (**E**–**H**). Honey reduces the wound-healing potential of MIA PaCa-2 and AsPC-1 cells. The brightfield mages were captured at 0, 24, and 48 h of incubation using Themo Fisher’s EVOSTM M7000 images system at 10X magnification objective lens; the magnification scale bar represents 275 µm. The wound closure (% ±SD) was calculated using GraphPad Prism, and the level of significance (control vs. 3% honey) was determined through a two-tailed unpaired *t*-test; ^ns^ *p* ≥ 0.05; *** *p* < 0.0005; **** *p* < 0.0001.

The cytotoxic effect of honey was initially investigated in four different pancreatic cancer cells, i.e., MIA PaCa-2, AsPC-1, Capan-2, and HPAF-II. The cells were treated with different concentrations of honey (0, 0.31, 0.63, 1.25, 2.50, 5.0, and 10.0%) for 24 h. As shown in Figure 2A, the MTT assay depicted that honey substantially inhibited the growth of these pancreatic cancer cells with IC_50_ values as low as 3.05, 4.62, 7.44, and 6.16% (*w*/*v*), respectively (Figure 2B). Based on these IC_50_ values, we chose MIA PaCa-2 and AsPC-1 cells for further studies. Firstly, the anti-cancer efficacy of this natural remedy was investigated through a colony formation assay to determine the potency of every single cell of a population to grow into a colony due to unlimited divisions. As a result, we reported that honey markedly restricted the clonogenic ability of MIA PaCa-2 and AsPC-1 cells when treated with low doses of honey (0.5 and 0.75%). However, untreated cells succeeded in giving rise to a significant number of colonies (Figure 2C and Figure 2D, respectively). We also investigated the therapeutic potential of honey on the invasiveness of these pancreatic cancer cells through a wound-healing assay. In this attempt, we found that honey markedly reduced the invasiveness of MIA PaCa-2 (Figure 2E,F) and AsPC-1 (Figure 2G,H) cells as depicted by compromised would/scratch healing after 24 and 48 h when compared to untreated cells. These initial findings clearly demonstrate the potent antiproliferative efficacy of honey and led us to further explore the molecular insights for the better management of pancreatic cancer through this natural remedy.

### 3.2. Honey Targets Ribosome Biogenesis to Achieve Potential Antiproliferative Effect against Pancreatic Cancer

**Figure 3:**
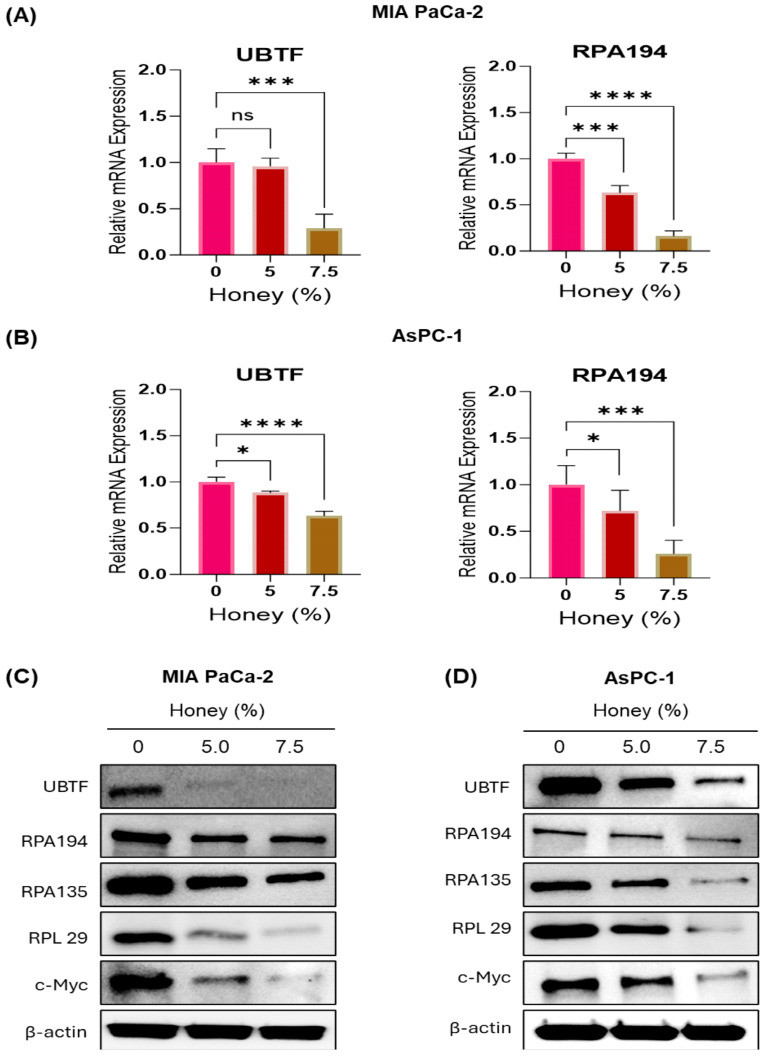
**Honey potentially targets ribosome biogenesis in human PanCa**. (**A**). Honey inhibits the mRNA expression of UBTF and RPA194 in MIA PaCa-2 cells (24 h). Data are represented as a fold of the untreated control ± SEM of three replicates of each dose of honey. Level of significance: ^ns^ *p* = 0.8658; *** *p* < 0.0009; **** *p* < 0.0001. (**B**). Honey inhibits the mRNA expression of UBTF and RPA194 in AsPC-1 cells (24 h). Data are represented as a fold of untreated control ± SEM of three replicates of each dose of honey. * *p* < 0.013; *** *p* < 0.0004; **** *p* < 0.0001. (**C**,**D**). Honey decreases the protein levels of key components of RNA Pol I pre-initiating complex of ribosome biogenesis, along with its regulator (c-Myc) and RPL29 in MIA PaCa-2 and AsPC1 cells (24 h).

Considering the inevitable role of ribosome biogenesis in the initiation, progression, advancement, and drug resistance in various cancers [8], we initially assessed the inhibitory effect of honey on the mRNA expression of key components of ribosome biogenesis through qRT-PCR analysis. In this attempt, honey exhibited a significant decrease in the mRNA expression of major molecular targets of ribosome biogenesis, i.e., UBTF and RPA194 in MIA PaCa-2 cells (Figure 3A). Briefly, honey reduced the expression of UBTF up to 0.96- and 0.29-fold in MIA PaCa-2 cells treated with 5 and 7.5% honey, respectively, when compared to the untreated control cells. The doses of honey were selected based on the cytotoxicity of honey against PanCa cells observed in the current study, as well as a previously published report [50]. Moreover, the expression of RPA194 was also suppressed to 0.63- and 0.16-fold in 5 and 7.5% honey-treated MIA PaCa-2 cells, respectively, when compared to the untreated control cells. A similar impact of honey on the mRNA expression of these key biomarkers of ribosome biogenesis, including UBTF and RPA194, was also observed in AsPC-1 cells (Figure 3B).

In addition to the mRNA expression, the impact of honey treatment on the proteins involved in ribosome biogenesis was investigated in MIA PaCa-2 and AsPC-1 cells. Honey substantially reduced the expressions of catalytic units of Pol I (RPA194 and RPA135) and its transcriptional activator (UBTF). Moreover, honey suppressed the expression of one of the key ribosomal proteins (RPL29) and c-Myc in both MIA PaCa-2 (Figure 3C and Supplementary Figure S1A) and AsPC-1 cells (Figure 3D and Supplementary Figure S1B). Please refer to Supplementary Figure S2 for full western blots.

**Figure 4:**
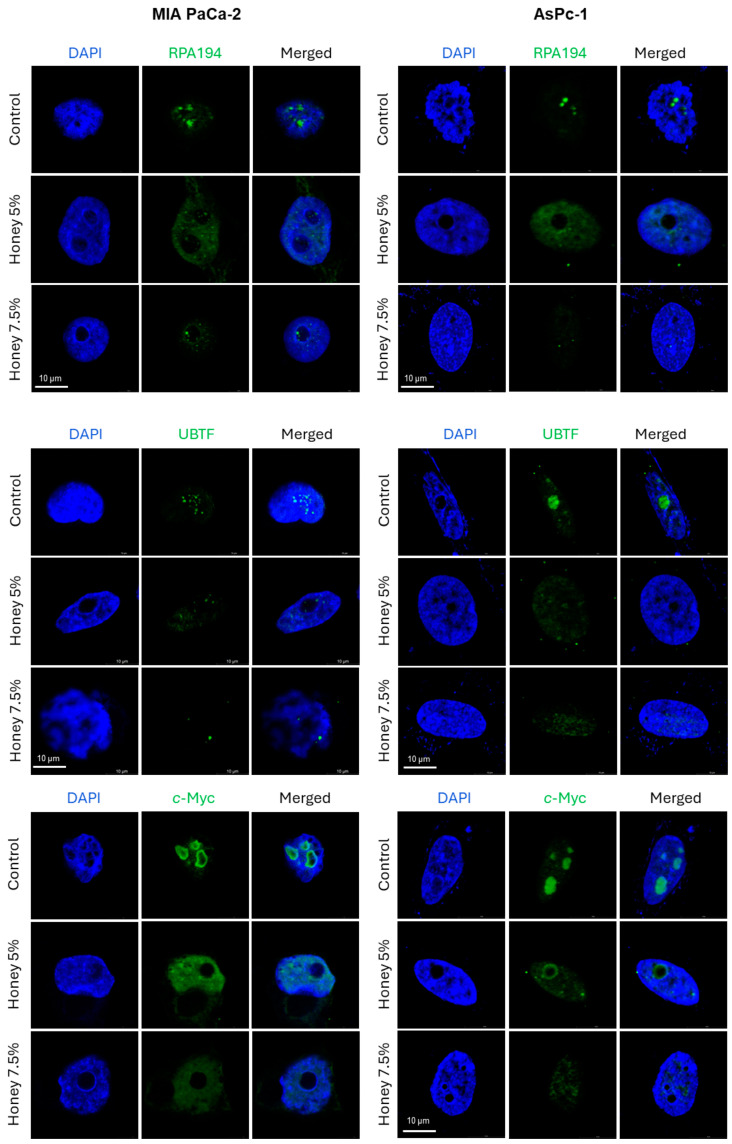
**Honey disrupts RNA Pol I subunits and c-Myc to modulate ribosome biogenesis in MIA PaCa-2 and AsPC-1 cells**. The images were acquired after 24 h of honey exposure through Leica STELLARIS STED & STELLARIS 8 STED Microscope. Magnification scale bar represents 10 µm.

Immunofluorescence (IF) results revealed that honey markedly disrupted the nucleolar RPA194 and UBTF in both MIA PaCa-2 and AsPC-1 cells (Figure 4). The treatment with honey also led to the disruption and delocalization of c-Myc, a well reckoned transcriptional activator of UBTF [51], from nucleolus into nucleus in both cell lines. These findings validate our hypothesis that honey exerts potent anti-cancer efficacy by targeting ribosome biogenesis in pancreatic cancer cells.

### 3.3. Honey Treatment Leads to the Suppression of Major Nucleolar Regulatory Proteins in Pancreatic Cancer Cells

**Figure 5:**
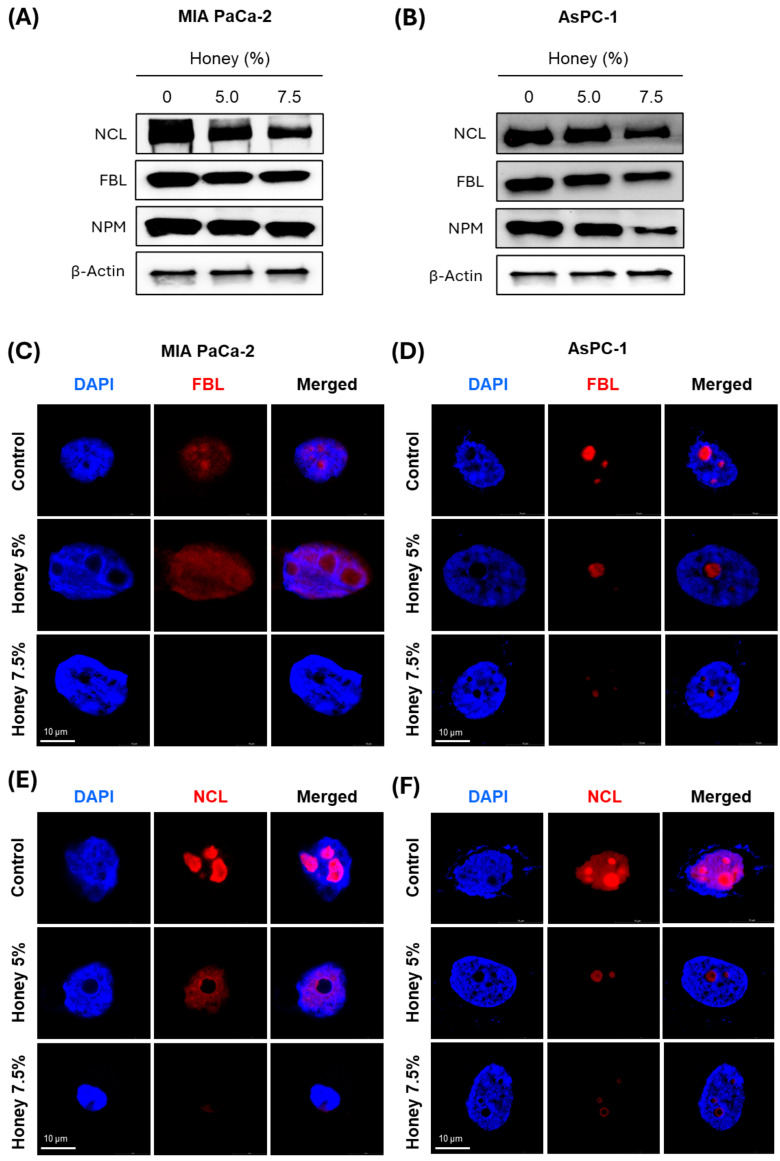
**Honey inhibits the expression of nucleolar proteins in PanCa**. (**A**,**B**). Effect of honey on protein levels NC, FBL, and NPM protein levels in MIA PaCa-2 and AsPC-1 cells, as determined using Western blot analysis after 24 h of honey exposure. Equal loading of protein in each lane was assessed by probing the blots with β-actin antibody. (**C**,**D**). Effect of honey on nucleolar localization of FBL (red) in MIA PaCa-2 and AsPC-1 cells, as determined using confocal microscopy after 24 h of honey exposure. (**E**,**F**). Effect of honey on nucleolar localization of NCL (red) in MIA PaCa-2 and AsPC-1 cells after 24 h of honey exposure. The images were acquired through Leica STELLARIS STED & STELLARIS 8 STED Confocal Microscope at a magnification scale bar of 10 µm.

There are various genes considered crucial in the context of nuclear genetics, cancer initiation, and advancement. Amongst these, nucleolin (NCL), fibrillarin (FBL), and nucleophosmin (NPM) are the most studied genes, and their overexpression has been correlated with the poor prognosis of various cancers [52,53,54]. These initial studies have established these nuclear/nucleolar proteins as preferred biomarkers for targeted therapy. Based on these reports, we assessed the effect of honey on the expression of these proteins in MIA PaCa-2 and AsPC-1 cells. Western blotting analysis showed that the expression of NCL, FBL, and NPM was markedly reduced after honey treatment in both MIA PaCa-2 (Figure 5A and Supplementary Figure S3A) and AsPC-1 (Figure 5B and Supplementary Figure S3B) cells when compared to corresponding untreated cells. Please refer to Supplementary Figure S4 for full western blots. IF analysis showed that FBL and NCL were localized in the nucleus, and honey treatment substantially decreased their expression in both cell lines (Figure 5C–F). These studies clearly indicated that honey leads to the disruption of major nuclear regulatory proteins to restrict the proliferation of pancreatic cancer cells.

### 3.4. Honey Induces Apoptosis in Pancreatic Cancer Cells

**Figure 6:**
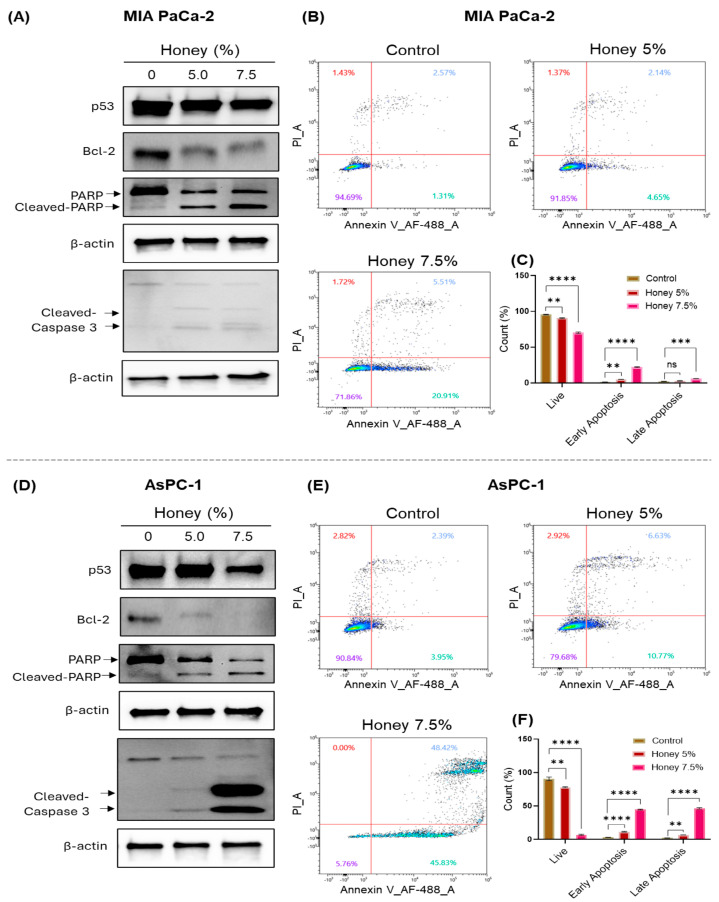
**Honey treatment induces apoptosis in PanCa cells**. (**A**). Effect of honey on protein levels of mutant p53, Bcl-2, caspase-3, and PARP in MIA PaCa-2 cells as determined using Western blot analysis after 24 h of honey exposure. Equal loading of protein in each lane was assessed by probing the blots with β-actin antibody. (**B**). Representative flow cytometry micrographs showing apoptosis in MIA PaCa-2 cells treated with honey for 24 h. The Annexin V/PI-stained cells were analyzed on SONY’ ID7000 Spectral Cell Analyzer. Three independent runs were recorded for each treatment group. (**C**). The bar graph represents quantification of live, early, and late apoptotic MIA PaCa-2 cells. The data in the bar graph represent the % count ± SEM of three replicates of each dose of honey. Level of significance: ^ns^ *p* = 0.4234; ** *p* < 0.0027; *** *p* < 0.0004; **** *p* < 0.0001. (**D**). Effect of honey on protein levels of mutant p53, Bcl-2, caspase-3, and PARP in AsPC-1 cells as determined using Western blot analysis after 24 h of honey exposure. Equal loading of protein in each lane was assessed by probing the blots with β-actin antibody. (**E**). Representative flow cytometry micrographs showing apoptosis in AsPC-1 cells treated with honey for 24 h. (**F**). The bar graph represents the quantification of live, early, and late apoptotic AsPC-1 cells. The data in the bar graph represent the % count ± SEM of three replicates of each dose of honey. Level of significance: ** *p* < 0.0035; **** *p* < 0.0001.

Following the nuclear stress, we aimed to investigate the impact of honey on apoptotic events through Western blotting and Annexin V-based flow cytometric analysis in MIA PaCa-2 and AsPC-1 cells. Honey treatment downregulated the expression of Bcl-2 and p53 (mutant form) in MIA PaCa-2 cells. Moreover, a significant increase in PARP and caspase-3 cleavage was observed in honey-treated MIA PaCa-2 cells when compared to untreated control cells (Figure 6A). Please refer to Supplementary Figure S5 for full western blots. These apoptotic events were further validated through Annexin V-based flow cytometry, which showed a decline in live population from 94.69% in control to 91.85% and 71.86% in 5 and 7.5% honey-treated cells, respectively. An increase in both early and late apoptotic populations was seen in honey-treated samples when compared to the untreated control MIA PaCa-2 cells (Figure 6B,C).

Similarly, honey exerted a similar suppressive impact on the expression of Bcl-2 and p53 (truncated form) in AsPC-1 cells, along with the remarkable increase in the cleavage of PARP and caspase-3 after 24 h of honey exposure when compared to the untreated cells (Figure 6D). Please refer to Supplementary Figure S5 for full western blots. On the other hand, the flow cytometric analysis revealed a marked decline in the live population from 90.84% in control to 79.68% and 5.76% in 5 and 7.5% honey-treated AsPC-1 cells, respectively. Interestingly, the AsPC-1 cells treated with 7.5% honey showed a noticeable hike in the early (45.83%) and late apoptotic (48.42%) populations (Figure 6E,F). These findings clearly show the potent anti-cancer efficacy of honey against pancreatic cancer.

### 3.5. Honey Treatment Leads to Cell Cycle Arrest in MIA PaCa-2 and AsPC-1 Cells

**Figure 7:**
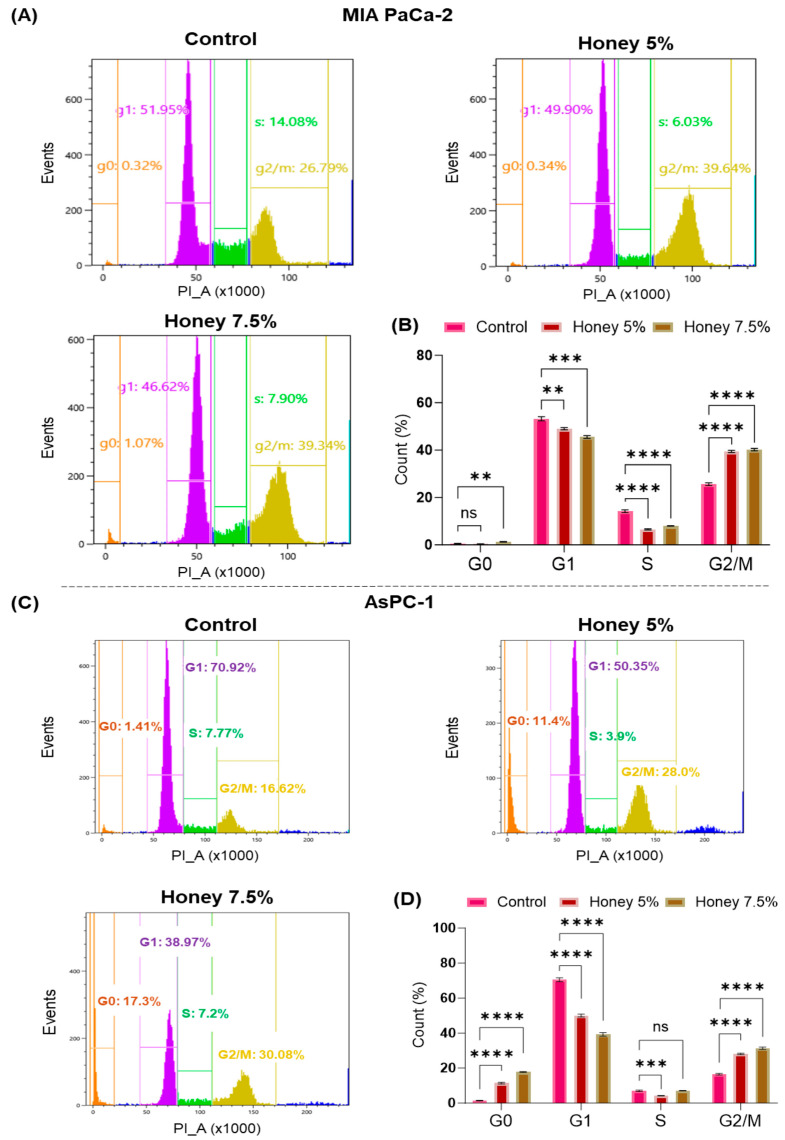
**Honey induces cell cycle arrest in PanCa cells**. (**A**). Effect of honey on cell cycle distribution in MIA PaCa-2 cells as determined by flow cytometric analysis after 24 h of honey exposure. (**B**). The bar graph represents the % distribution of the MIA PaCa-2 cells in the G0, G1, S, and G2M phases. The level of significance: ^ns ^*p* = 0.5470; ** *p* < 0.0037; *** *p* < 0.0002; **** *p* < 0.0001. (**C**). Honey arrests the cell cycle in the G0 and G2/M phases of AsPC-1 cells after 24 h of honey exposure. (**D**). The bar graph represents the % distribution of the AsPC-1 population in the G0, G1, S, and G2M phases. The level of significance: ^ns^ *p* = 0.9260; *** *p* < 0.0009; **** *p* < 0.0001.

To further explore the mechanism behind the antiproliferative potential of honey, we performed flow cytometric cell cycle analysis in MIA PaCa-2 and AsPC-1 cells. In this attempt, honey treatment led to a G2/M phase arrest in MIA PaCa-2 cells (G2/M: 39.34% at 7.5% honey) when compared to untreated control cells (G2/M: 26.79%). Moreover, a significant decline was also observed in G1 (52% in control vs. 46.62% in 7.5% honey-treated samples) and S phase (14.08% in control vs. 7.90% in 7.5% honey-treated samples) populations in these cells (Figure 7A,B). Unlike MIA PaCa-2, the treatment with honey in AsPC-1 led to a significant arrest in the G0 population at 5 and 7.5% honey (11.45% and 17.30%, respectively) when compared to untreated control AsPC-1 cells (G0: 1.41% only). Interestingly, significant arrest in a G2/M phase (G2/M: 30.08% at 7.5% honey) was also reported in honey-treated AsPC-1 cells when compared to control cells (G2/M: 16.62%) (Figure 7C,D). These results validate our above-mentioned antiproliferative potential of honey in pancreatic cancer cells.

## 4. Discussion

Currently available therapies for PanCa, either alone or in combination, barely improve the 5-year overall survival, and the limited availability of early diagnostic strategies has further challenged the therapeutic management of PanCa. In the current study, we aimed to uncover the antiproliferative efficacy of honey as an alternative therapy by targeting ribosome biogenesis for the management of PanCa. In this attempt, we initially reported that honey markedly inhibits the proliferation of PanCa cell lines MIA PaCa-2, AsPC-1, HPAF-II, and Capan-2, with the lowest IC_50_ value in MIA PaCa-2 and AsPC-1 cells, hence, being chosen for further studies. The cytotoxic effect of honey on these cell lines was well in agreement with a previously published report that demonstrated the anti-cancer effect of honey against prostate cancer cells when used up to 10% *w*/*v* [50]. Furthermore, honey also impacted the clonogenic ability and invasiveness of MIA PaCa-2 and AsPC-1 cells, as evidenced by colony formation and wound healing assays, respectively. These interesting findings led us to uncover the molecular mechanism behind the remarkable antiproliferative potential of honey. In this order, honey demonstrated a dual inhibitory impact on ribosome biogenesis by suppressing the mRNA expression of UBTF and RPA194 in both cell lines.

Interestingly, such dual targeting of RPA194 and UBTF has not been reported in the case of any specific inhibitor/s. Even recently discovered specific RPA194 inhibitors, BMH-21 and CX-5461, did not exhibit any effect on UBTF [9]. Moreover, honey exposure significantly downregulated the protein expression of UBTF, RPA194, and RPA135 in both cell lines. Such downregulation of UBTF, RPA194, and RPA135 at the protein level by any specific therapeutic agent has not been reported yet. In contrast, BMH-21 (1 µM) induces the proteasomal degradation of RPA194, but at the same time, it also causes segregation of nucleolar structures [55]. In addition to these key subunits of RNA Pol-I and its transcription factor (UBTF), the expression of c-Myc was also suppressed at the protein level after the cells were exposed to honey. This downregulation of c-Myc expression by honey might be correlated with the downregulation of UBTF, as c-Myc had previously been demonstrated to activate the rDNA transcription via directly interacting with the UBTF promotor sequence [51]. Furthermore, the expression of RPL29, one of the key RPs overexpressed in various cancer types [15], was also downregulated by honey in PanCa cells. IF studies also confirmed the suppressive impact of honey on key ribosomal proteins, as nuclear disruption and translocation of RPA194, UBTF, and c-Myc was observed in honey-treated MIA PaCa-2 and AsPC-1 cells. A similar nucleolar segregation of UBTF was observed after BMH-21 treatment in a previous study [55].

The impact of honey on the regulation of ribosome biogenesis might be attributed to the presence of bioactive metabolites (refer to the introduction section), which might act individually or synergistically to target various components of ribosome biogenesis. However, we could not find any report demonstrating the regulatory effect of any of these bioactive metabolites on ribosome biogenesis except for gallic acid [56]. Gallic acid has been shown to suppress rRNA transcription via reactive oxygen species-mediated activation of Lysine-specific histone demethylase 2A (KDM2A) in breast cancer MCF-7 cells. In contrast, gallic acid exposure did not activate KDM2A in non-tumorigenic MCF10A cells [56]. Overall, these findings strongly advocate the potential impact of honey on ribosome biogenesis for the management of PanCa.

The disruption of the nucleolar structures and alterations in nucleolar protein localization and dynamics have been established as the hallmark of ribosome biogenesis blockade therapy. In this line, NCL, FBL, and NPM are the most studied nucleolar proteins, and their overexpression has been correlated with the poor prognosis of various cancers [52,53,54]. NCL has recently been reported to regulate tumor growth and angiogenesis in PanCa while targeting NCL through a pseudopeptide N6L, a specific inhibitor of NCL, resulting in normalized angiogenesis and reduced the population of regulatory T-cells and myeloid-derived immunosuppressor cells in PanCa. Moreover, therapeutic targeting of NCL also stimulated the tumor-infiltrated T lymphocytes, impaired cancer-associated fibroblast, and reprogrammed the tumor microenvironment via suppression of interleukin-6 in PanCa [57]. On the other hand, FBL, a well-established nucleolar methyltransferase that catalyzes the methylation of rRNA and RPs, has been linked to the enhanced resistance of cancer cells to DNA damage caused by DNA crosslinkers, i.e., cisplatin, camptothecin, and etoposide. FBL also stimulates the homologous recombination-mediated DNA repair in cancer [58].

Similarly, the overexpression of NPM has also been associated with the poor prognosis of PanCa as it stimulates glucose uptake and leads to a metabolic switch from aerobic glycolysis to oxidative phosphorylation in NPM-overexpressing BxPC-3 cells. In contrast, the knockdown of NPM stimulated the expression of fructose-1, 6-bisphosphatase 1 (FBP1) in AsPC-1, Panc-1, and BxPC-3 PanCa cells [59]. Herein, we found that honey markedly downregulates the expression of NCL, FBL, and NPM at the protein level in a dose-dependent manner in MIA PaCa-2 and AsPC-1 cells. In contrast, even being a specific inhibitor of ribosome biogenesis, BMH-21 did not show any effect on the protein level of NCL, FBL, and NPM [55]. These findings were further validated through IF investigations, which clearly demonstrated that honey treatment leads to the nucleolar translocation/disruption of NCL and FBL in MIA PaCa-2 and AsPC-1 cells. This translocation of NCL and FBL by honey markedly affected the proliferation and survival of cancer cells in our study.

On the other hand, p53 (encoded by the *TP53* gene), a tumor suppressor protein, acts as a major barrier against cancer initiation and progression [60]. However, genetic aberrations in TP53 are very common in cancer, and the extent of these *TP53* mutations can be imagined by the fact that approximately 75–90% of PanCa patients harbor *TP53* mutations [61], and there is no standard-of-care therapy for the treatment of mutated *TP53*. MIA PaCa-2 and AsPC-1 are well known to possess mutated and truncated *TP53*, respectively [62], and the extent of mutant p53 has also been associated with the gemcitabine resistance in PanCa.

Interestingly, in the current study, honey was found to downregulate the expression of mutated *TP53* in both cell lines, which further signifies the potential of honey to induce apoptosis in PanCa cells. Moreover, the process of apoptosis is very complex and tightly regulated by the coordination of distinct mediators, including Bcl-2, which negatively impacts apoptotic events [63]; therefore, it is considered the preferred target in the management of cancer. In this context, we assessed the impact of honey on Bcl-2 expression and found that honey markedly reduces the expression of Bcl-2 at the protein level. Similarly, cleavage of PARP is considered a useful hallmark of apoptotic cell death. Caspases, namely caspase-3 and caspase-7, play a crucial role in the cleavage of PARP. Upon cleavage, PARP loses its functionality and ultimately leads to the suppression of DNA repair [64]. In the same line, we also reported a substantial PARP cleavage in both cell lines upon treatment with honey. Caspase-3, also known as executioner caspase, plays a crucial role in the terminal stages of apoptosis [65]. However, caspase-3 is activated by the action of caspase-8, caspase-9, and caspase-10 [66,67]. Nonetheless, cleavage of caspase-3 is a hallmark of terminal apoptosis [65,68]. Together with the PARP, noticeable cleavage was observed in caspase-3, as well as in honey-exposed PanCa cells. These studies were followed by the Annexin V-mediated apoptosis assay, which further validated the here-mentioned findings that honey potentially induces apoptosis in PanCa cells. To sum up, honey exerts a potent ability to induce apoptosis via the downregulation of mutant TP53 and Bcl-2, along with the cleavage of PARP and caspase-3 in PanCa cells.

On the other hand, dysregulation of the cell cycle due to compromised activity of cyclins and cyclin-dependent kinases leads to the development of various cancers, particularly PanCa [69,70]. Interestingly, mutations in CDKN2A have been strongly correlated with about 45–60% of PanCa cases [69,71]. Therefore, therapeutic modulation of the cell cycle is considered an important approach to treat such cancers. In this order, honey treatment was found to induce G2/M arrest in MIA PaCa-2 and dual G0 and G2/M arrest in AsPC-1 cells, signifying its ability to regulate cell cycle checkpoints and subsequent cell death.

## 5. Conclusions

Our results strongly suggest that honey has the potential to induce apoptosis and inhibit the growth and invasive phenotypes of PanCa cells by targeting various components of ribosome biogenesis. We speculate that honey could be utilized as an adjuvant to ongoing chemo/immunotherapy regimens for the prevention and treatment of human PanCa. Further detailed studies are warranted to investigate its anti-cancer potential using appropriate pre-clinical mouse models of PanCa.

## Figures and Tables

**Table 1:** Primers used for qRT-PCR analysis are appended below.

S. No.	Gene	Primer Sequence	
		Forward (5′-3′)	Reverse (5′-3′)
1.	UBTF	CAAAACCACCGAATCACACA	TGTCAATGTACGGAACTTCCT C
2.	RPA194	GAA GTTGCCAGAGGAAGT G	CTGGGTACTTGTCCATCATTA G
3.	β-actin	ACAGCCTGGATAGCAAGG	CACCAACTGGGACGACAT

## Data Availability

The data generated in the current study has been published in this article as well as supplied as supplementary information that can be downloaded from the online version of this article from the Journal’s website.

## Supplementary Materials

The following supporting information can be downloaded at https://www.mdpi.com/article/10.3390/cancers16193431/s1, Figure S1: (A,B): Quantitation of western blots for the proteins involved in ribosome biogenesis in MIA PaCa-2 cells and AsPC-1; Figure S2: (A,B): Full western blots of proteins involved in ribosome biogenesis in MIA PaCa-2 cells and AsPc-1 cells. Figure S3: (A,B): Quantitation of western blots for the proteins involved in nucleolar organization in MIA PaCa-2 and AsPC-1 cells; Figure S4: (A,B): Full western blots of proteins involved in nucleolar organization in MIA PaCa-2 cells and AsPc-1 cells. Figure S5: (A,B): Western blots of proteins involved in apoptosis in MIA PaCa-2 and AsPC-1 cells.
