# Supplementary material for: Honey Targets Ribosome Biogenesis Components to Suppress the Growth of Human Pancreatic Cancer Cells
Source: Cancers (Basel). 2024 Oct 9;16(19):3431. doi: 10.3390/cancers16193431 (PMC11475701; doi:10.3390/cancers16193431)
Supplement: Supplementary file 1 [file cancers-16-03431-s001.zip › cancers-3218679-supplementary.pdf]

Supplementary Material

Journal: Cancers (ISSN: 2072-6694)

Research Article

Honey Targets Ribosome Biogenesis Components to Suppress the growth of Human Pancreatic Cancer Cells

Aun Ali Bangash [1,2,#], Sahir Sultan Alvi [1,2,*], Muhammad Ali Bangash [1,2,#], Haider Ahsan [1,2], Shiza Khan [1,2], Rida Shareef [1,2], Georgina Villanueva [1,2], Divyam Bansal [3], Mudassier Ahmad [1,2], Dae J. Kim [1,2], Subhash C. Chauhan [1,2], Bilal Bin Hafeez [1,2,*]

[1] South Texas Center of Excellence for Cancer Research, School of Medicine, University of Texas Rio Grande Valley, McAllen, 78504, Texas, United states of America; aun.bangash01@utrgv.edu (A.A.B.); muhammad.bangash01@utrgv.edu (M.A.B.); haider.ahsan01@utrgv.edu (H.A.); shiza.khan01@utrgv.edu (S.K.); rida.shareef01@utrgv.edu (R.S.); georgina.villanueva01@utrgv.edu (G.V.); mudassier.ahmad@utrgv.edu (M.A.); dae.kim@utrgv.edu (D.J.K.); subhash.chauhan@utrgv.edu (S.C.C.).

[2] Department of Medicine and Oncology ISU, Division of Immunology and Microbiology, School of Medicine, University of Texas Rio Grande Valley, McAllen, 78504, Texas, United states of America

[3] Rice University, Houston, Texas, United states of America; db65@rice.edu

*Correspondence: sahir.alvi@utrgv.edu (S.S.A.); Tel.: +1(956)-658-4507; bilal.hafeez@utrgv.edu (B.B.H.); Tel.: +1-(901)-608-2182

# These authors share equal authorship.

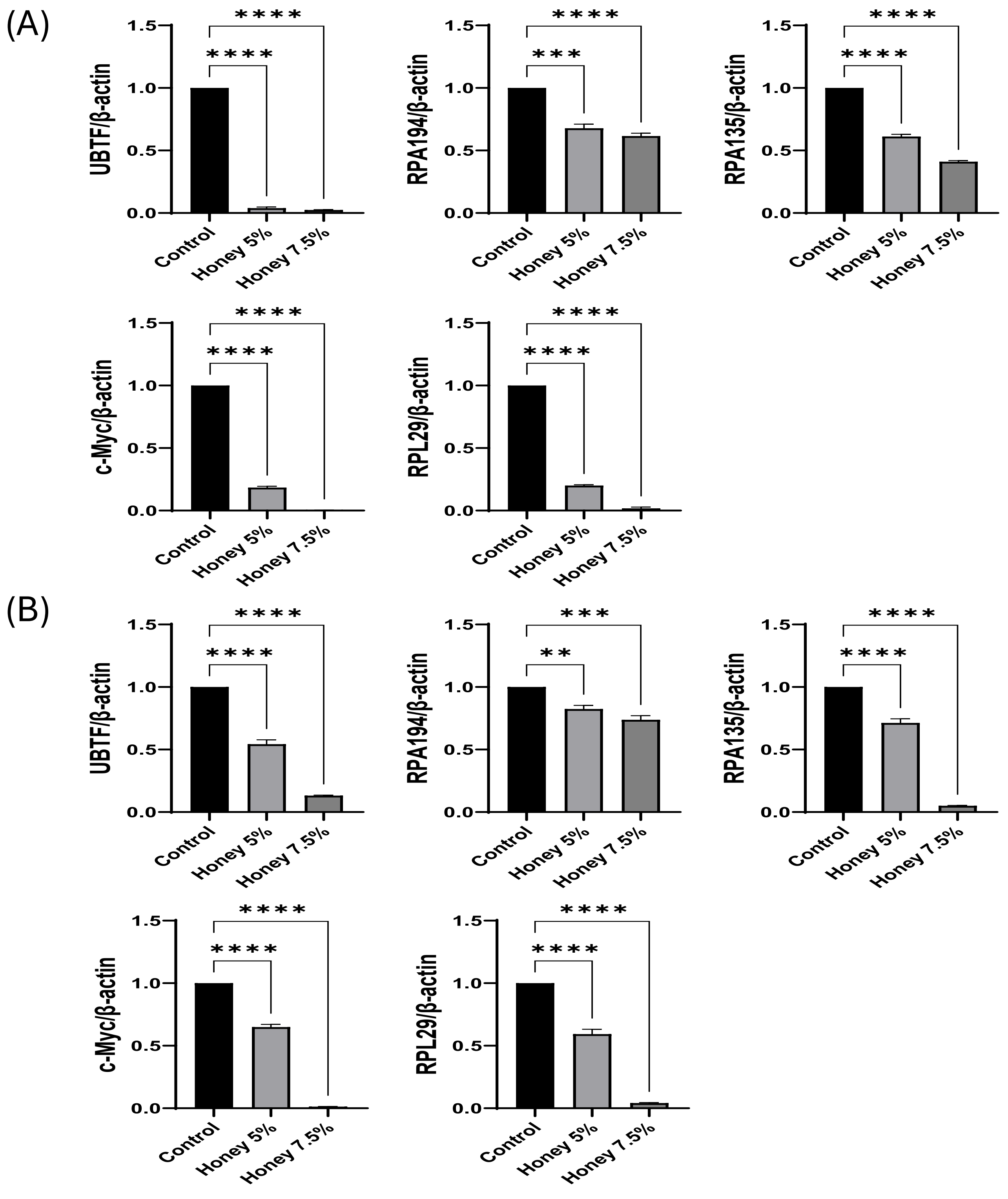

Supplementary Figure S1: (A); Quantitation of western blots for the proteins involved in ribosome biogenesis in MIA PaCa-2 cells. The graphs were quantitated using ImageJ software. The statistical analysis was done using GraphPad Prism 10.0.0. The comparisons were done using One way ANOVA followed by Dunnett's multiple comparison test. ***p=0.0001, ****p<0.0001. **(B)**; Quantitation of western blots for the proteins involved in ribosome biogenesis in AsPC-1 cells. The statistical analysis was done using GraphPad Prism 10.0.0. The comparisons were done using One way ANOVA followed by Dunnett's multiple comparison test. **p=0.0045, ***p=0.0001, ****p<0.0001.

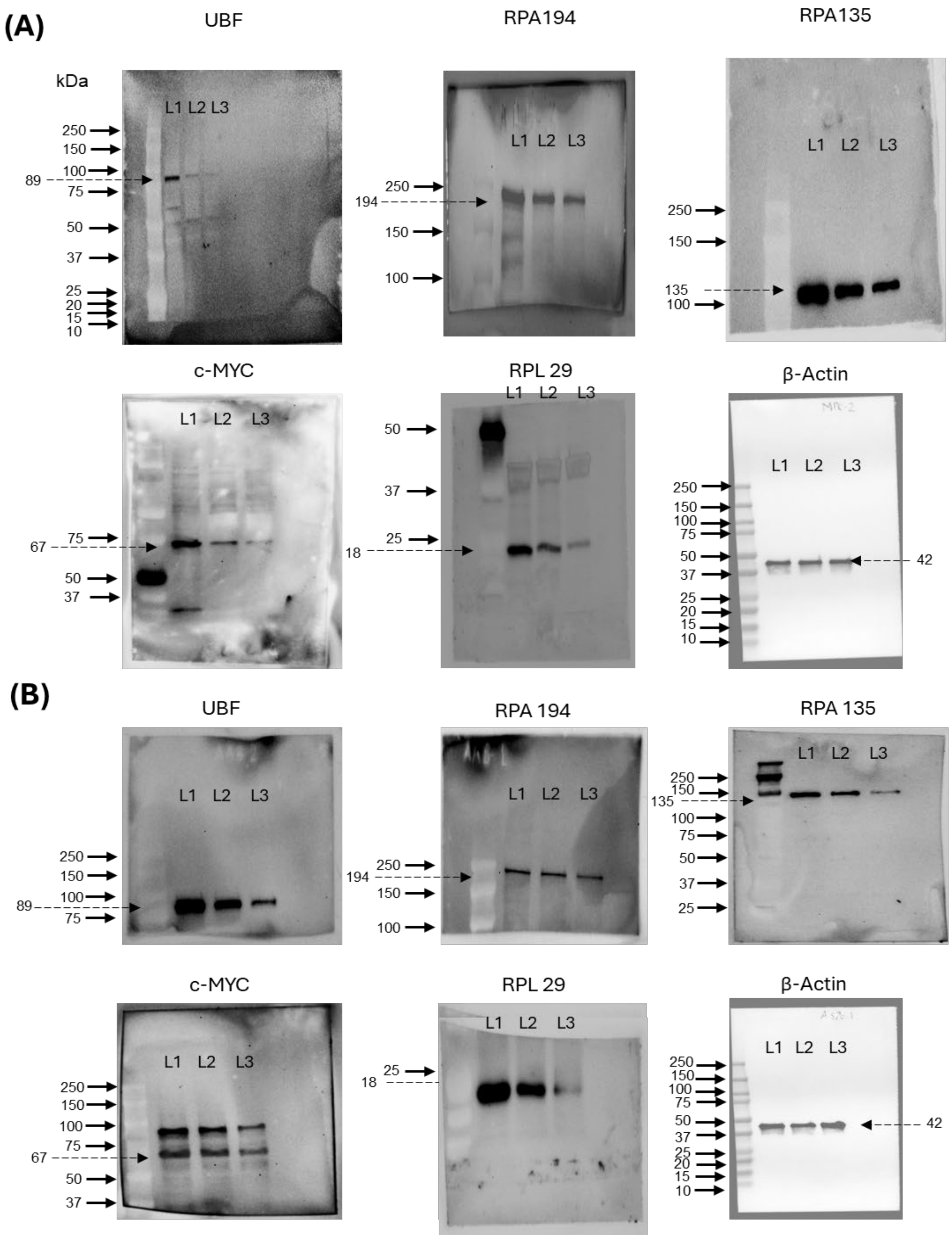

Supplementary Figure S2: (A); Western blots of proteins involved in Ribosome biogenesis in MIA PaCa-2 cells. **(B);** Western blots of proteins involved in Ribosome biogenesis in AsPC-1 cells. Lane L1, L2, and L3 represent control, honey 5% , and honey 7.5%, respectively, for both cell lines.

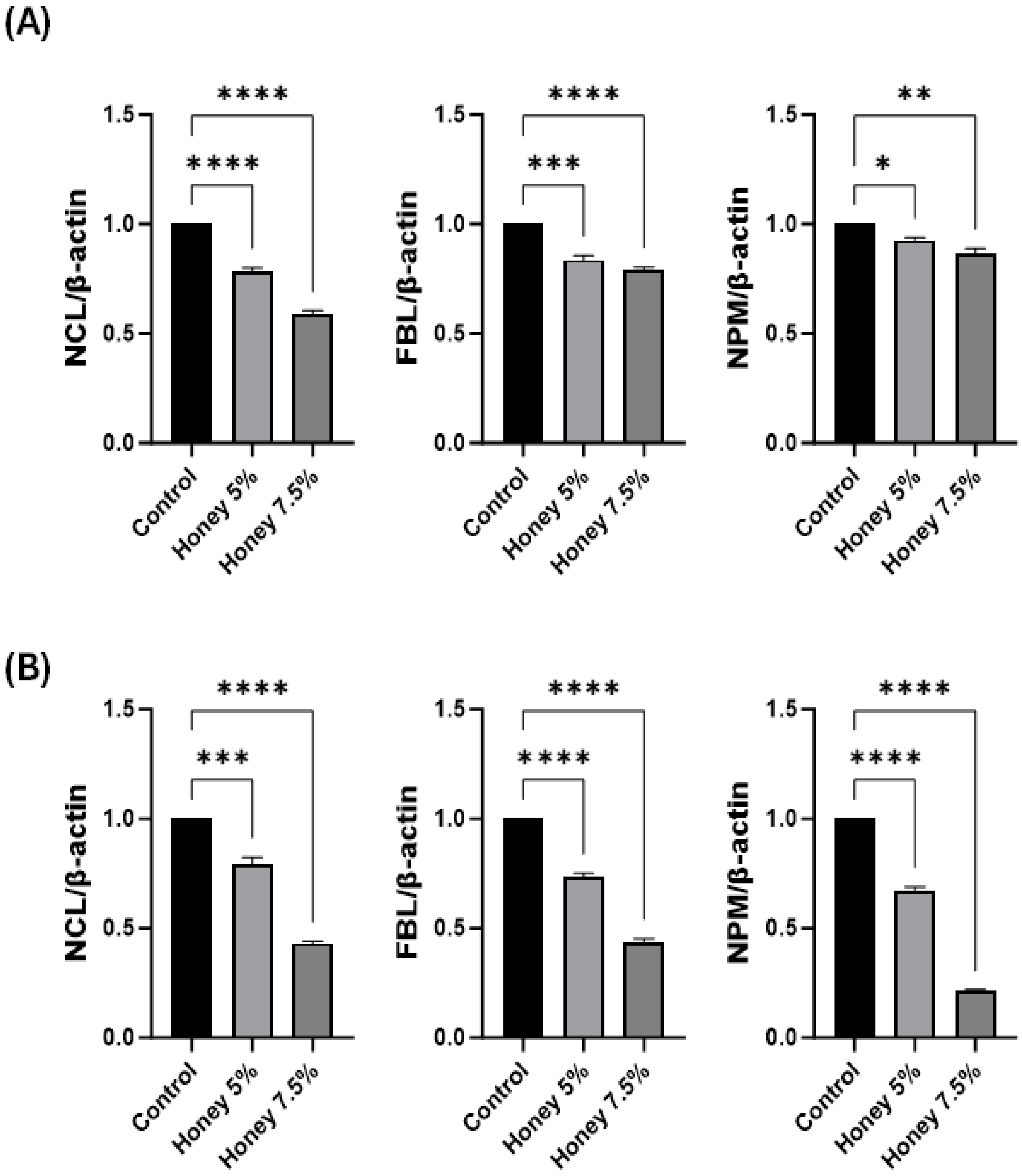

Supplementary Figure 3: (A); Quantitation of western blots for the proteins involved in nucleolar organization in MIA PaCa-2 cells. The graphs were quantitated using ImageJ software. The statistical analysis was done using GraphPad Prism 10.0.0. The comparisons were done using One way ANOVA followed by Dunnett's multiple comparison test. *p=0.0265, **p=0.0045, ***p=0.0001, ****p<0.0001. **(B);** Quantitation of western blots for the proteins involved in nucleolar organization in AsPC-1 cells. The statistical analysis was done using GraphPad Prism 10.0.0. The comparisons were done using One way ANOVA followed by Dunnett's multiple comparison test. ***p=0.0001, ****p<0.0001.

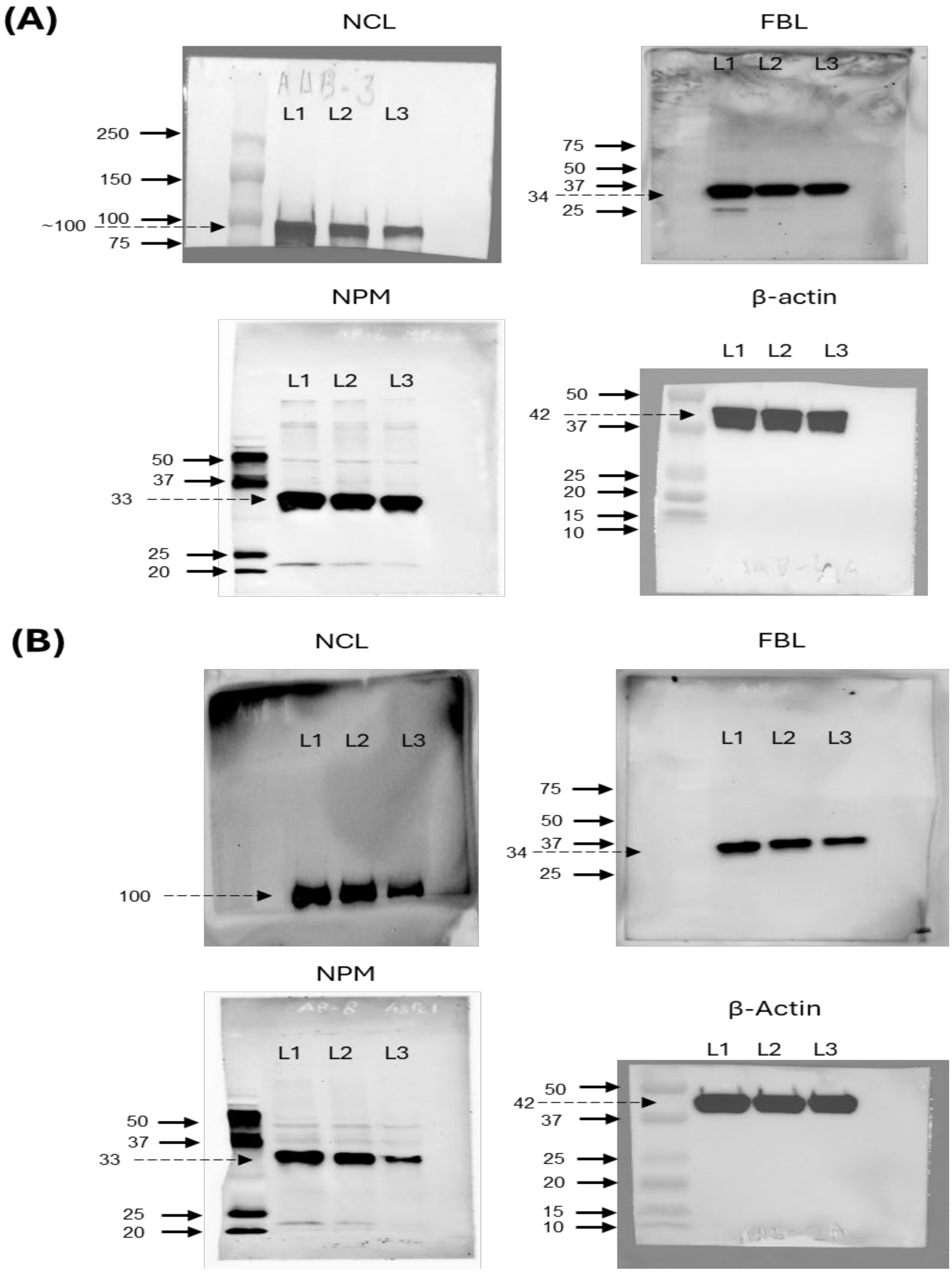

Supplementary Figure S4: (A); Western blots of proteins involved in nucleolar organization in MIA PaCa-2 cells. **(B);** Western blots of proteins involved in nucleolar organization in AsPC-1 cells. Lane L1, L2, and L3 represent control, honey 5% , and honey 7.5%, respectively, for both cell lines.

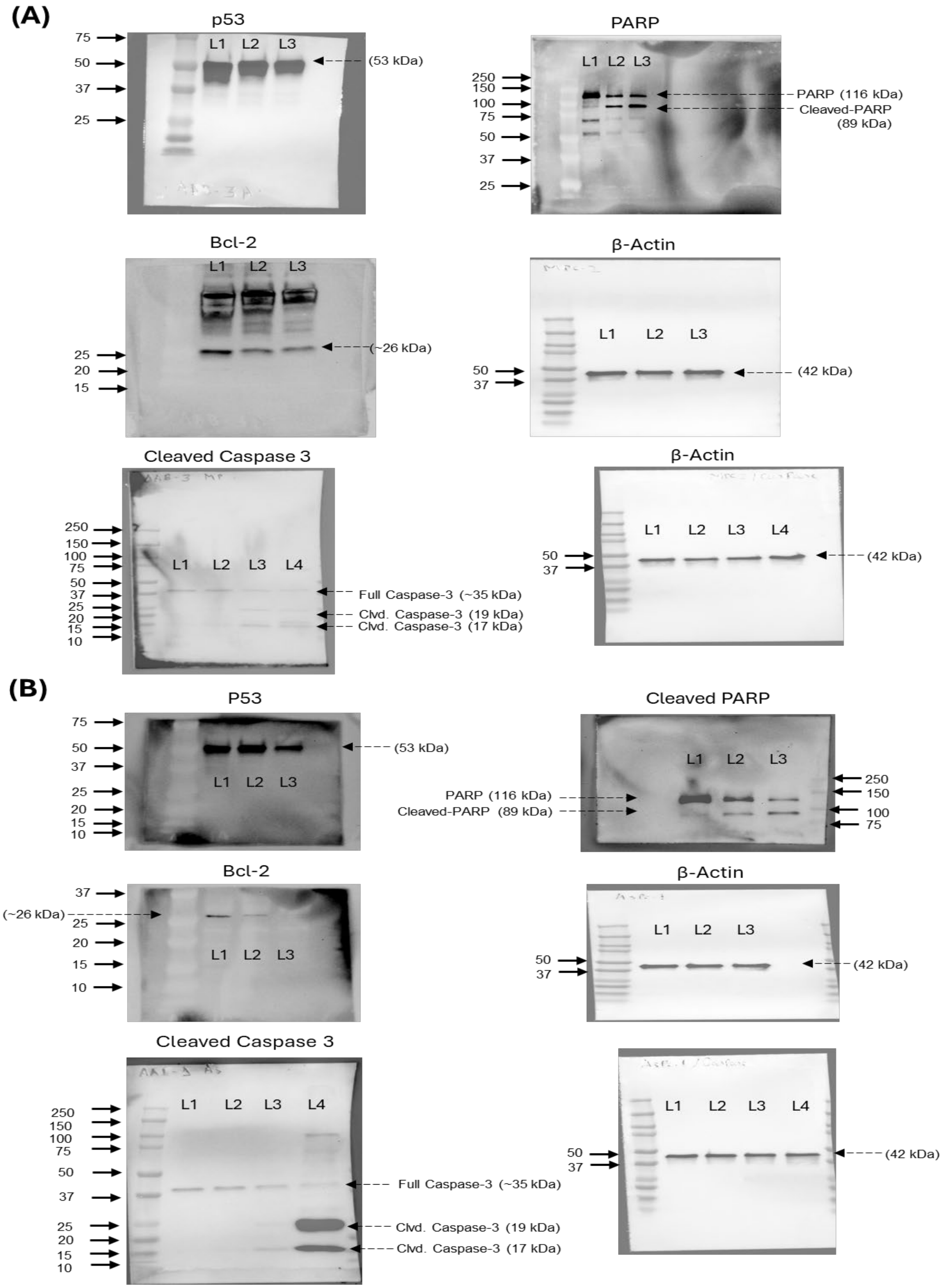

Supplementary Figure S5: (A); Western blots of proteins involved in apoptosis in MIA PaCa-2 cells. **(B);** Western blots of proteins involved in apoptosis in AsPC-1 cells. Lane L1, L2, and L3 represent control, honey 5% , and honey 7.5%, respectively, for all proteins except caspase-3 where lane L1, L2, L3 and L4 represent Control 1, Control 2, honey 5% , and honey 7.5%, respectively.
